# PALPABLE: enhanced bending stiffness and proprioception in soft actuators for laparoscopic palpation

**DOI:** 10.3389/frobt.2026.1832079

**Published:** 2026-07-06

**Authors:** Christos Vasileios Lousis, Myrto Inglezou, Ilias Zournatzis, Georgios Violakis, Panagiotis Polygerinos

**Affiliations:** 1 Department of Mechanical Engineering, School of Engineering, Control Systems and Robotics Laboratory (CSRL), Hellenic Mediterranean University, Heraklion, Greece; 2 AI Innovation Center, University of Essex, Colchester, United Kingdom; 3 Bendabl SMPC, Athens, Greece; 4 Department of Industrial Design and Production Engineering, School of Engineering, University of West Attica, Athens, Greece

**Keywords:** fiber optic, pneumatic, shape reconstruction, soft actuator, stiffness optimization, surgical robotics, syringe pump

## Abstract

The assessment of tissue properties via direct palpation is essential for localizing abnormalities during surgery. However, Minimally Invasive Surgery (MIS) eliminates this tactile feedback, creating a critical sensory gap. To address this, the EU Horizon project PALPABLE is developing novel fiber-optic sensing modalities for stiffness assessment. Successful integration of this technology requires an articulation interface that balances high maneuverability with the structural stability necessary for deflection-free tissue palpation. We present a single-degree-of-freedom (DOF) soft silicone actuator integrated with a modular, bioinspired passive stiffening element. Mechanical loading tests, motion tracking, and Finite Element Method (FEM)-guided design iterations validate the stiffening strategy and actuator performance. The design achieves a substantial increase in bending stiffness, improving the stiffness–pressure response by 8-fold relative to the unstiffened actuator under critical loading (normal force at a 90° bend). Furthermore, we developed a compact, custom pneumatic syringe pump with closed-loop pressure control to ensure precise, safe operation in surgical environments. By combining embedded fiber-optic sensing with machine-learning models, the system achieves free-space full-shape reconstruction with approximately 1% error. This work delivers a compact, load-capable, and sensorized soft actuator tailored for PALPABLE’s probe manipulation requirements, enabling safe, controllable, and data-rich tissue palpation in confined anatomical spaces.

## 1 Introduction

The inherent compliance and morphological adaptability of soft bending actuators have led to their rapid adoption across many applications. This trend is particularly evident in surgical robotics (Rodrigue and Kim, 2024; Sarker et al., 2025), where systems have been proposed for minimally invasive access, physiological monitoring, and tissue manipulation (Ataollahi et al., 2013; Gorissen et al., 2018; Zhang Y. et al., 2023).These developments address key design constraints such as stiffness, miniaturization, anatomical safety (Cianchetti et al., 2013; Shi et al., 2024) and shape sensing (Juliá Wise et al., 2025; An et al., 2024). Among these, achieving sufficient stiffness under load while maintaining accurate shape sensing has become a major focus in recent literature.

Reliable operation under external loading is a key performance requirement for soft bending actuators and remains a persistent challenge in soft robotics (Yang et al., 2018). To address this, various stiffness-modulation strategies have been explored, broadly categorized as active or passive approaches (Dou et al., 2021). Active methods typically rely on external stimuli to alter the actuator’s mechanical properties. Examples include antagonistic pneumatic or hydraulic chambers (Wang et al., 2021), vacuum- or pressure-based jamming structures (Wall et al., 2015), tendon-driven tensioning mechanisms (Shiva et al., 2016), and smart-material cores activated by thermal, electrical, or magnetic fields (Liao et al., 2021; Liu et al., 2020; Gaeta et al., 2023). While these approaches enable precise and tunable stiffness control, they often increase system complexity, resulting in bulky hardware and complicating fabrication and control.

Passive strategies, in contrast, incorporate structural elements that intrinsically modulate actuator mechanics without necessitating external energy input. These approaches are divided mainly into expansion restrictors and stiffening cores. Expansion restrictors mitigate radial deformation in pressurized chambers by using rings, fibers, or mesh reinforcements, improving bending efficiency and programming motion profiles (Polygerinos et al., 2015; Sauter et al., 2022). However, such designs often require multi-step casting or expose reinforcement materials that compromise surface smoothness. Alternatively, embedded stiffening cores allow bending while increasing resistance to external loads. Examples include passive granular inserts (Li et al., 2017), elongation-limiting elements (Yap et al., 2015; Licher et al., 2025), and elastic geometric features that constrain deformation modes to enable directional stiffening (Ochoa et al., 2025). Although these passive methods improve stiffness, reproducibility, and modularity, they often require higher operating pressures to induce bending.

While stiffness modulation and proprioceptive sensing are central challenges for soft actuators in general, surgical applications impose additional constraints that further restrict the design space. Conventionally, shape reconstruction relies on analytical models like Piecewise Constant Curvature (PCC) or Cosserat rod theory (WebsterIII and Jones, 2010; Grazioso et al., 2019). However, these models require precise physical parameters and often fail to account for non-linearities like hysteresis or manufacturing defects, leading to significant error propagation (Sefati et al., 2021). Consequently, research has shifted toward data-driven strategies that map sensor data directly to task space (Ha et al., 2023; Shekari and Moosavian, 2025) or even, incorporating actuator states to improve accuracy (Zhang A. et al., 2023).

This work presents the design, fabrication, and control of a soft bending actuator with proprioception for laparoscopic use, compatible with PALPABLE’s requirements and its custom pneumatic supply unit. A key innovation is a modular bio-inspired passive stiffening spine that enhances stiffness in all load directions and provides soft locking at a 90° bend while maintaining flexibility. Its geometry and performance are optimized through bench-top mechanical testing, finite-element modeling, and statistical analysis. The actuator is integrated with a pneumatic syringe pump and a closed-loop control system for precise and reliable operation, while inscribed Fiber Bragg Grating (FBG) sensors enable real-time shape sensing and provide proprioceptive feedback.

The work is organized as follows: Section 2 describes the actuator design and fabrication; Section 3 details the stiffening spine’s design iterations, physical testing, and FEM-assisted optimization; Section 5 presents the pump design and control; Section 6 covers shape sensing and actuator control; and Section 7 discusses the system’s performance and concludes the work.

## 2 Actuator design and fabrication

### 2.1 Requirements analysis and system architecture

**FIGURE 1:**
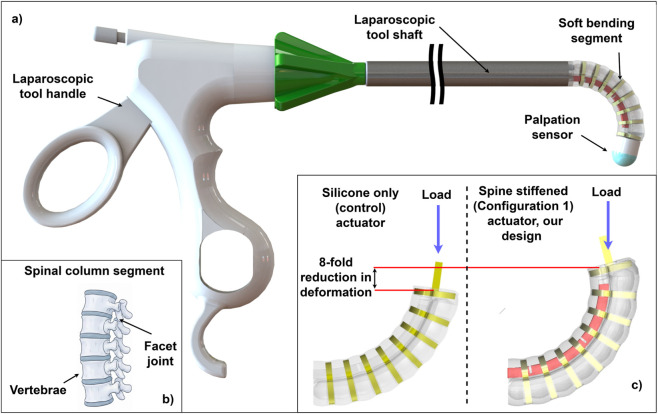
**(a)** Illustration of the PALPABLE system including, from left to right, the laparoscopic handle providing rotational and bending control; the 12 mm diameter laparoscopic shaft; the soft bio-inspired bending segment featuring FBG-based fiber optic proprioception and pneumatic actuation; and the hemispherical stiffness-sensing probe tip. **(b)** The spinal structure featuring vertebrae and facet joints that provide the bio-inspired basis for the stiffening segment’s design. **(c)** Illustrative deflection comparison between a baseline silicone-only actuator and the spine-stiffened actuator developed in this work.

The actuator enabling the full use of PALPABLE’s palpation sensing probe must satisfy several application-specific constraints. The probe (Figure 1a), consists of a compliant hemispherical membrane surrounding a central base. It contains embedded optical fibers in a crossed pattern and utilizes Fiber Bragg Gratings (FBG) technology. During palpation, the membrane deforms according to tissue compliance, and the resulting changes in optical transmission encode force and displacement, enabling local tissue stiffness estimation. Achieving this requires, the actuator reliably orienting and rotating the probe inside the human body, while sustaining palpation forces with patient safety and comfort as the foremost considerations.

The actuator is designed as a compact, single-degree-of-freedom, monolithic soft bending segment tailored for minimally invasive surgical environments. While previous soft laparoscopic systems have individually demonstrated advancements in stiffness modulation, shape sensing, miniaturization, modularity, atraumatic design, sterilizability, and simplified fabrication (Gerboni et al., 2015; Decroly et al., 2021; Dai et al., 2023; Juliá Wise et al., 2025), no single system has successfully integrated these often conflicting requirements into a unified device. The integration of the PALPABLE sensing probe further constrains the operational space, necessitating an actuator that is simultaneously compact, smooth-profiled, modular and sterilizable, while sustaining palpation loads without compromising bending performance.

To address these multifaceted requirements, in collaboration with clinicians from the European Association of Endoscopic Surgery (EAES), the actuator’s constraints were defined (Table 1). The system must be compatible with minimally invasive laparoscopic diagnostic surgery, limiting the outer diameter to 15 mm, in correspondence to one of the available standard trocar port sizes. As laparoscopic tools inherently provide 360° axial rotation, the actuator is required to bend in a single direction, reaching up to 90° for navigation. Based on empirical data, collected from clinical experts of the (EAES), ensuring safe tissue interaction and meaningful tactile feedback, requires that the actuator withstands a 1 N palpation load applied only when straight (0°) or fully deflected (90°). Additionally this requirement fully covers the probe’s operating range. Real-time monitoring of the actuator’s bending angle necessitates the integration of embedded sensing. Due to the probe’s complexity, the actuation system must allow easy removal, replacement, and reusability, introducing the need for accessible lumens, quick release and connect attachment interfaces, and use of sterilizable materials. Finally, to ensure tissue safety, smooth and body-safe geometries and mechanical robustness under overload conditions are required.

**TABLE 1:** PALPABLE’s actuator design constraints in accordance to EAES collaborator’s directions.

Specification	Requirement addressed
1 N load at normal direction with <1 mm tip deflection at $0^{o}$ and $90^{o}$	Enables palpation, shape retention under load
Modular attachment of end effector and optical fiber routing	Probe re-usability, interchangeable sensing probes
Body safe materials	Patient safety, sterilization, (ISO-10993–5) compliant silicone
Modular incorporation of shape sensing subsystem	Re-usability, casting simplification
$90^{o}$ bending with $360^{o}$ rotation	Full utilization of probe sensing capabilities
Simple single casting actuator fabrication	Cost efficiency, manufacturability
Real-time shape sensing	Usability, safe manipulation, feedback control

A silicone body of 14.8 mm diameter was selected, containing a semicircular pneumatic chamber optimized for bending efficiency (Moutousi and Polygerinos, 2024). Because laparoscopic tools inherently allow 360° axial rotation, the actuator only needs to bend in a single plane, reaching up to 90° to cover the probe’s operational workspace. This baseline configuration is designated as the Control and serves as a benchmark for the stiffened configurations presented in Section 3.

### 2.2 Structural features for stiffness, smoothness, and modularity

#### 2.2.1 Radial expansion constraint rings

**FIGURE 2:**
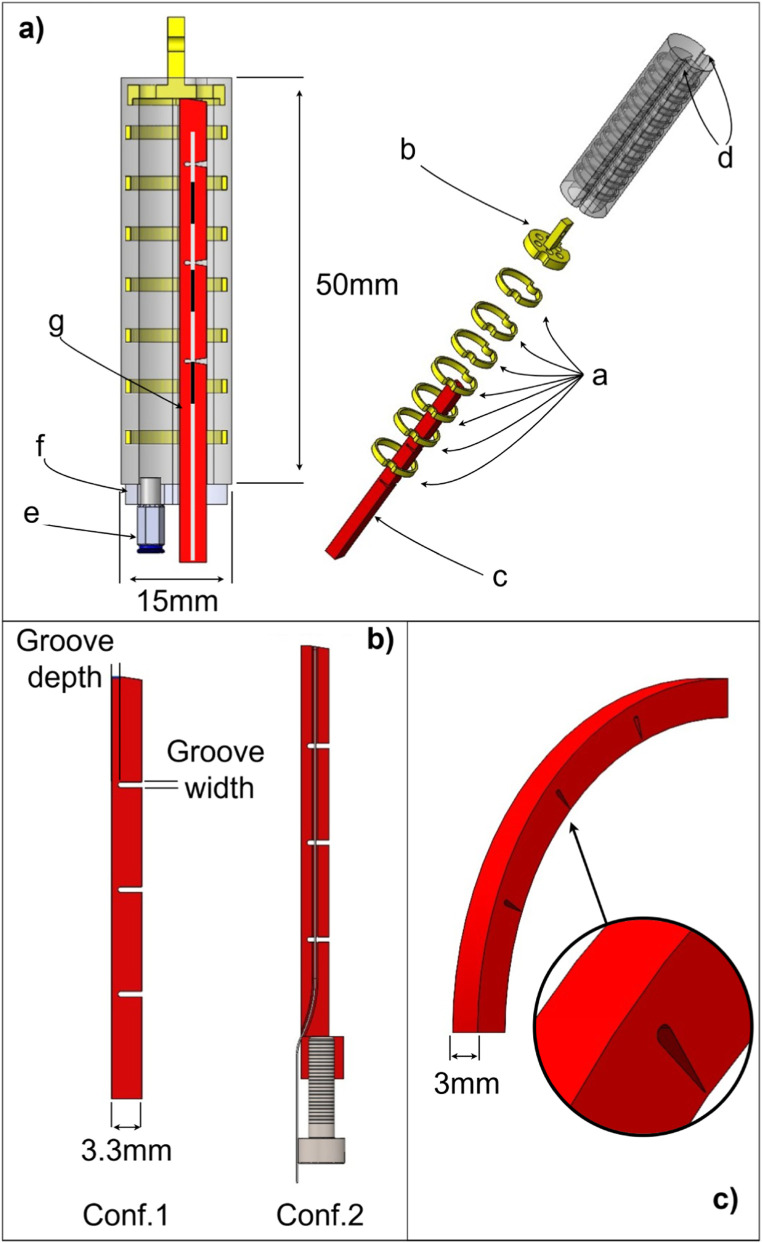
**(a)** Depiction of the pneumatically driven, silicone-based actuator comprised of: (a) silicone embedded radial expansion constraint rings, (b) distal end sensor attachment disk, (c) modular stiffening spine, (d) external to the silicone body lumens utilized by the distal end sensing probe, (e) pneumatic fitting, (f) proximal shaft attachment disk, and (g) FBG based shape sensing optic fiber housed in the spine. **(b)** Spine Configuration 1 and 2 untensioned showcasing the optimization parameters Groove depth, Groove width and thickness. **(c)** Spine Configuration 1 tensioned showing vertebral contact that results in increased stiffness.

To achieve the required load-bearing capacity, seven radial expansion-constraint rings were integrated along the actuator body (Figure 2a). Their number and spacing were determined by the 15 mm outer diameter constraint and the need to maintain a single-step casting process, avoiding excessive manufacturing complexity. Preliminary prototyping showed that fewer rings caused localized ballooning of the pneumatic channel, while higher ring density required significantly greater actuation pressures to achieve similar bending. The seven-ring configuration therefore provides an optimal trade-off between reinforcement density, bending efficiency, and manufacturing feasibility, consistent with reinforcement strategies reported in prior soft actuator designs (Nguyen et al., 2019; Zournatzis et al., 2023).

**FIGURE 3:**
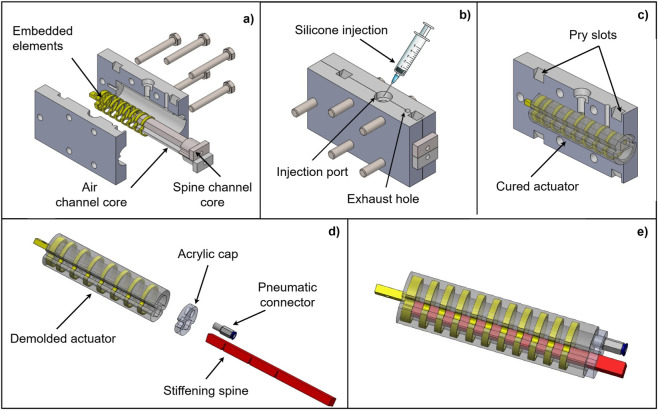
**(a)** Embedded elements and cores placed inside the 2-part mold while it is still open. The mold is then closed and secured with screws. **(b)** De-gassed silicone being injected with the syringe in the mold and cured in a heated chamber. **(c)** Opened mold, with the air and spine channel cores removed. **(d)** Actuator assembly; the acrylic end cap is glued with the pneumatic connector attached to it and the spine inserted in its corresponding cavity. **(e)** Ready to use actuator.

To ensure a smooth, monolithic cylindrical profile compliant with sterilization protocols (Chemical sterilisation, Ethylene oxide and other low temperature methods), the constraint rings are fully encapsulated within the silicone matrix. Achieving this encapsulation in a single casting step required a design modification: each ring features two small mounting grooves. These interfaces allow the rings to be suspended within the mold cavity, facilitating unobstructed silicone flow and eliminating the multistep fabrication procedures typically required for actuators with embedded reinforcement (Polygerinos et al., 2015; Galloway et al., 2013; Su et al., 2019). Because the rings are fully encapsulated, they can be digitally fabricated using any material capable of withstanding sterilization processes without compromising the actuator’s external bio-compatibility. Consequently, polylactic acid (PLA) was selected for this application.

#### 2.2.2 External lumens for routing probe fibers

The mounting grooves utilized for ring positioning were extended longitudinally to form external lumens spanning the actuator’s length (Figure 2a). This design feature provides a direct routing path for the probe’s optical fibers (or auxiliary cabling), facilitating integration without requiring partial disassembly of either the probe or the actuator. Post-casting, the lumens are sealed with a thin layer of specialized silicone adhesive (Sil-Poxy^TM^) to occlude any ring mounting gaps. The optical fibers of the probe’s membranes are then routed through the lumens and subsequently secured via interference fit or localized adhesive bonding.

#### 2.2.3 Modular probe attachment disk

A perforated disk, mirroring the outer profile of the constraint rings, is embedded at the distal end of the actuator. Its protruding mounting interface provides rapid, rigid, and accessible attachment of the PALPABLE’s probe. Consistent with the reinforcement rings, the disk is primarily encapsulated in silicone; the exposed attachment point is shielded by the probe body upon assembly, ensuring safety. Accordingly, this component was also digitally fabricated using PLA.

#### 2.2.4 Bio-inspired stiffening spine

The design process yielded three distinct actuator variants: the Control, Configuration 1, and Configuration 2. Beyond the Control actuator, to further augment load capacity and minimize deflection under palpation forces, a bio-inspired insertable stiffening spine was introduced. Detailed in Section 3, this component features transverse grooves inspired by vertebral facet joints (Figure 1b). These geometric features facilitate compliance in bending while significantly enhancing stiffness across multiple loading directions. Figure (Figure 2b), illustrates the spines of Configuration 1, which employs the foundational grooved spine architecture, and Configuration 2, which integrates a high-stiffness, pre-tensioned tendon. This tendon is routed through the spine to connect the distal and proximal ends of the actuator and spine, respectively, thereby constraining axial elongation. Both variants are modular, removable, and fully isolated from the external environment during use. Fabricated via digital methods in TPU 95A, the spines enable rapid manufacturing while ensuring bio-compatibility and sterilizability.

#### 2.2.5 Embedded shape sensing

To enable the position and shape tracking of the actuator, a standard single core, single mode telecommunications optical fiber (SMF-28e, Corning Glass Inc., USA) featuring three inscribed Fiber Bragg Gratings (FBGs) was integrated into the actuator’s stiffening spine. Being situated there ensures its free bending, undisturbed by the silicone bodies non-linear and high friction behavior All FBGs were third order gratings of 1.6 mm length with a spacing of 3.5 mm between them (edge to edge distance). All three FBGs were fabricated using the point by point technique and a 1030 nm 4W femtosecond laser (LightConversion ltd., Lithuania) focused through a ×63 oil immersion objective and scanned via high precision translation stages (Aerotech Ltd., USA). To facilitate non-permanent attachment and ensure reusability, a central lumen was incorporated within the spine, through which the fiber is routed.

### 2.3 Fabrication process

The fabrication for all three actuator variants followed a unified workflow with minor configuration-specific adaptations. The axial constraint rings, attachment disk, and removable internal cores, along with the tendon for the pre-tensioned configuration, were positioned inside a two-part mold (Figure 3a), shared by all actuator configurations. The mold allows suspension of embedded elements, via mounting grooves located on the rings, ensuring accurate alignment during casting. Once assembled, the mold is filled with degassed silicone and cured in an oven at 45 °C for 45 min (Figure 3b). Following curing, the actuator was de-molded and the internal cores are pulled out to form the pneumatic chamber, the central spine channel and the external lumens (Figure 3c). An acrylic end cap is bonded to the actuator’s proximal end using silicone adhesive with properties matching the cast silicone, providing an airtight seal and a mounting interface for a pneumatic push-fit connector (Figure 3d). Aside from the removable cores and optional stiffening elements, all configurations share the same fabrication steps and mold architecture, enabling consistent manufacturing results while accommodating design variations.

## 3 Spine design testing and optimisation

The Control actuator (i.e., without stiffening spine), despite providing sufficient support when aligned with the normal direction (0°), was unable to sustain the required loads at full deflection (90°) (Section 3.1). While increasing the stiffness of the silicone body could mitigate this limitation, it would result in substantially higher operating pressures and reduced compliance. Given the requirements for simplicity, manufacturability, and predictable behavior, a passive mechanical reinforcement was adopted. Drawing inspiration from the facet joints found in vertebrate spines (Figure 1c), a bio-inspired stiffening element, designated as the spine, was developed.

The spine comprises a flexible beam segmented by transverse grooves (Figure 2c). These grooves define controlled bending zones that facilitate low-resistance flexion while preserving the compressive stiffness of the “vertebra”. As the actuator approaches its maximum bending angle, adjacent segments engage in mechanical contact, producing a marked increase in stiffness along the palpation vector. Concurrently, friction between the spine and the inner channel wall, induced by the air channel’s deformation during pressurization, restricts axial elongation. Collectively, these mechanisms enhance load-bearing performance across multiple bending configurations without imposing significant pressure increment.

Space for the spine was limited to a 5 × 6 mm cavity due to the actuator’s outer diameter, air channels, and embedded components. To prevent tearing, uneven casting, and air-channel rupture, testing required a minimum silicone wall thickness of 0.2 mm around all embedded elements, which dictated the spine’s maximum cross-sectional area. Modularity requirements, specifically the need to recover the optical sensing probe, prevents permanently casting the spine in place. Furthermore, casting a separate lumen directly into the actuator body was unfeasible due to space limits, manufacturing unreliability, and potential friction when inserting the optic fiber. Therefore, the spine served as the only viable host for a lumen used for shape-sensor routing.

### 3.1 Spine testing and optimization

**FIGURE 4:**
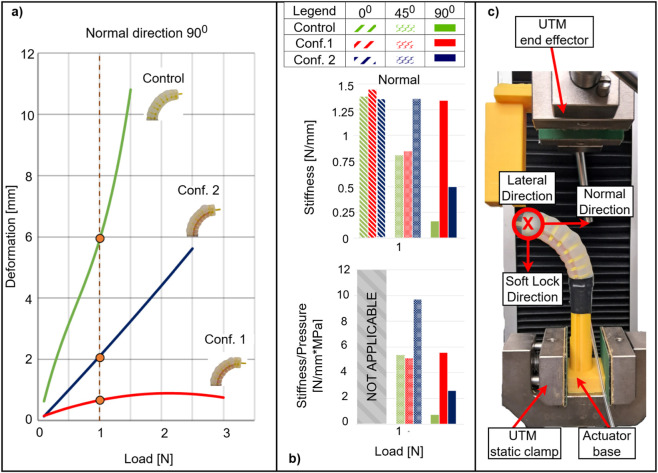
**(a)** Deformation behaviour across the loading range of the 3 configurations during UTM compression testing at a 90° angle under load in the normal direction; all 3 configurations bended at a 90° angle. **(b)** Stiffness and pressure efficiency charts of the three configurations in multiple angles under different loading directions. **(c)** UTM testing arrangement and loading directions.

To identify the optimal architecture for further optimization, a comparative experimental series was conducted. Each configuration was tested using a Universal Testing Machine (UTM) at three bending angles (0°, 45°, and 90°), across three loading vectors: normal, lateral, and soft lock (Figure 4c). The normal vector is perpendicular to the probe’s membrane and lies within the actuator’s bending plane, while the lateral vector is perpendicular to that plane. The soft lock vector, perpendicular to the plane defined by the normal and lateral vectors, was assessed exclusively at 90°. This is because the significant stiffness enhancement relies on the vertebral contact mechanism, which engages most effectively at full deflection. To account for manufacturing variability, two spines of each configuration were tested on two separate actuators, alongside two Control samples. To ensure material consistency, all components were digitally fabricated using freshly opened, desiccated filaments from a single manufacturer.

**FIGURE 5:**
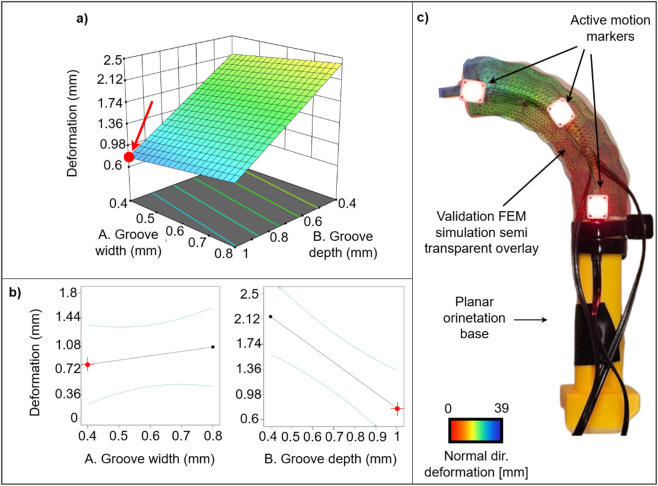
**(a)** Box-Behken response surface for deformation minimization, showcasing the optimal design point regarding groove width and depth along with the design’s predicted deformation. **(b)** DOE correlation curves for groove width and depth demonstrating each parameter’s effect on deformation, with the other fixed at its optimal value. **(c)** Motion tracking arrangement of the Control actuator bent at 90° using 3 active motion markers, overlayed with the semi-transparent FEM result of the Control actuator under the same loading condition.

Each actuator was bonded to a base featuring three planar surfaces oriented at 0°, 45°, and 90° to serve as fixed indexing references (Figure 5c). The base was secured to the UTM, and the actuator was pressurized until its distal end aligned with the vertical compression end-effector, corresponding to the specific load case. The base design incorporates an aperture to facilitate the rapid exchange of spines. Finally, the UTM end-effector was driven vertically to measure the applied load and resulting actuator deformation (Figure 4c).

The actuation pressure required for each actuator–angle pair is summarized in (Table 2). Configuration 2 demonstrated a clear performance advantage over the Control, reducing the pressure required to reach 90° by 13%. Conversely, Configuration 1 necessitated a modest 8% increase in pressure. These disparities underscore the necessity of jointly evaluating structural stiffness and actuation efficiency when comparing the two spine configurations.

**TABLE 2:** Actuation pressures for all configuration-bending angle combinations.

Actuator type	0°	45°	90°
Control	0 kPa	150 kPa	220 kPa
Configuration 1	0 kPa	165 kPa	240 kPa
Configuration 2	0 kPa	140 kPa	190 kPa

#### 3.1.1 Stiffness evaluation

In the normal loading direction, the actuators were compared at the 1N load threshold. All three variants displayed largely linear stiffness behavior, with the exception of the Control at 90°. This linearity is a favorable characteristic that simplifies stiffness tuning for future applications. The normal direction tests highlighted the most significant performance gains provided by the spines, validating their intended design function. Most notably, Configuration 1 achieved an 800% increase in stiffness compared to the Control, at 90°.

The lateral load-bearing capacity of all actuators was inherently lower, consequently, comparative evaluations were conducted at a 0.2 N load threshold. Consistent with the results in the normal direction, the stiffness profiles remained predominantly linear. Both Configuration 1 and Configuration 2 displayed superior stiffness compared to the Control, demonstrating improvements ranging from 500% to 820% depending on the bending angle.

In the soft lock direction, both spine configurations withstood a buckling load exceeding double that of the Control actuator. Notably, Configuration 2 consistently exhibited approximately half the deformation of Configuration 1, representing a 690% increment relative to the Control (Figure 4b).

Overall, both spine architectures yielded substantial performance gains compared to the Control actuator. Configuration 1 and Configuration 2 performed comparably across most load-angle combinations, with each excelling in distinct scenarios. The observed performance variation can be attributed to the axial elongation inherent in Configuration 1, which alters the effective lever arm relative to the actuator’s base and modifies the spine’s bending geometry, thereby impeding consistent vertebral contact. While this elongation occasionally reduces structural stiffness due to suboptimal vertebral engagement, the concurrent increase in internal pressure provides compensation. This results in a performance trade-off that varies across specific loading vectors and bending angles. Additionally all loading experiments were extended to at least double the deformation at 1N or at least 2N (depending on the configuration and loading direction), to ensure the actuator wont fail under higher palpation forces (Figure 4a).

#### 3.1.2 Energy evaluation

Although structural stiffness remains the primary performance metric for the PALPABLE system, the three actuator configurations achieve target bending angles at different actuation pressures. To assess comprehensive efficiency, a second criterion, the stiffness-to-pressure ratio, was employed. This metric quantifies the actuation “cost” of stiffness in terms of energy consumption and pneumatic hardware requirements, thereby serving as a critical indicator for practical applications.

In the normal loading direction, both configurations outperform the Control, demonstrating significant efficiency gains, most notably, Configuration 1 achieved a 635% improvement over the Control at 90° . Similarly, in the lateral direction, improvements were observed relative to the Control, with Configuration 2 exhibiting a 155% increase over the baseline at 90° . In the soft lock direction, efficiency was significantly enhanced, with Configuration 2 outperforming the Control by 255%. Overall, the gains in actuation efficiency exceeded the proportional improvements in stiffness (Figure 4b). This confirms that both spine configurations provide substantial reinforcement while requiring either minimal additional pressure (Configuration 1) and lower actuation pressure (Configuration 2) compared to the Control. The ability of Configuration 2 to reduce pressure while improving stiffness is attributed to the inextensible tendon; by opposing the force tending to elongate the actuator, it creates a restorative moment. As the tendon follows the spine’s contour during bending, this moment amplifies the bending action. Consistent with the stiffness analysis, neither configuration proved universally superior, as suitability is contingent upon specific application requirements. Configuration 1 was selected for further optimization, as it successfully satisfied the critical design criterion of <1 mm deformation under a 1 N load at 90° bending.

### 3.2 FEM modeling

Numerical modeling of the actuator incorporating Configuration 1 was performed using SIMULIA Abaqus FEA and structured into two distinct phases: validation and optimization. The validation phase utilized the specific spine geometry from the experimental testing to ensure the model accurately replicated the physical system’s behavior in both free-space bending and palpation loading scenarios. Subsequently, the optimization phase employed Response Surface Methodology (RSM) to determine the spine geometry that minimizes deformation under a 1 N load at a 90° bending angle.

The validation phase demonstrated that the numerical model exhibits high fidelity to the experimental setup, achieving a tip position accuracy of 0.9 mm during free-space bending. Under normal loading conditions, the deformation error relative to the UTM data was limited to 0.6 mm. The silicone actuator body (Dragonskin 30, Smooth-on Inc.) and the TPU 95A spine were defined using hyperelastic material models, whereas the PLA components were modeled as linear elastic; all domains were discretized using C3D4 tetrahedral elements. The specific material parameters are detailed in (Table 3). Mesh convergence was verified by iteratively halving the element size until the variation in tip displacement fell below 5%. An encastrate boundary condition was applied to the proximal end of the assembly (encompassing both silicone and spine). The simulation was set to dynamic-explicit and the actuation achieved via cavity pressure, while the normal direction load was applied as a distributed nodal force acting perpendicularly to the actuator’s distal face.

**TABLE 3:** Material properties used in FEM simulations.

Element	Parameters	Source
Dragonskin 30	Mass density: 1080 kg/ $m^{3}$ Yeoh $2^{\text{nd}}$ order coefficients: C1 = 0.11, C2 = 0.07, D1 = 0, D2 = 0	Marechal et al. (2021)
TPU 95	Mass density: 1220 kg/ $m^{3}$ Yeoh $3^{\text{rd}}$ order coefficients: C1 = 1.2376, C2 = 0.9855, C3 = 0.1581, D1 = 0, D2 = 0, D3 = 0	Reyes et al. (2024)
PLA	Mass density: 1240 kg/ $m^{3}$ Young’s modulus: 3500 MPa Poisson’s ratio: 0.35	Provided by vendor

### 3.3 Position tracking of the actuator

While the UTM measurements quantified deformation under external loading, accurate modeling further required a precise correlation between actuation pressure and bending angle, alongside the kinematic tracking of key structural points. These parameters were captured using a high-fidelity motion capture system (IMPULSE X2E, PhaseSpace, CA, USA) tracking three active LED markers with positional accuracy of 20 μ m and a sampling rate of 960 Hz. The markers were mounted at equal spacing along one of the actuator’s external lumens: the first at the proximal base, the second at the mid-point, and the third at the distal tip (Figure 5c). The proximal marker served as a static reference frame, isolating the relative displacement of the medial and distal markers. The experimental protocol involved tracking the Configuration 1 assembly during pressurization up to 90° (240 kPa) in 5 kPa increments, with a 5-s dwell time at each pressure step to ensure steady-state measurement. Validation of the FEM model was carried out using the final step of the experimental protocol that considered a pressure level of 240 kPa. During the 5-s dwell time a mean value of 100 points was calculated around the median of the captured vector $\left(P_{\exp},X_{d,exp},Y_{d,exp}\right)$ , where $P_{\exp}$ is the pressure measurement at each timestamp, and $\left(X_{d,exp},Y_{d,exp}\right)$ are the spatial coordinates of the LED marker mounted at the distal end, in respect to the coordinate system defined by the LED marker mounted at the proximal base. Given these measurements, a mean value was calculated to describe the stabilized state of the max pressurization step as $\left({\bar{P}}_{\exp},{\bar{X}}_{d,exp},{\bar{Y}}_{d,exp}\right)$ . Subsequently, the average error of the position accuracy compared to the Finite Element Model was calculated as $\sqrt{{\left({\bar{X}}_{d,exp}-X_{d,sim}\right)}^{2}+{\left({\bar{Y}}_{d,exp}-Y_{d,sim}\right)}^{2}}=0.9mm$ , when ${\bar{P}}_{\exp}=P_{\mathrm{sim}}=240kPa$ , where the annotation sim refers to the simulated measurements calculated at the mesh node that corresponds to the distal end in respect to the coordinate system defined by the mesh node at the proximal base.

### 3.4 Spine optimization

Following the validation of the FEM, the actuator–spine system was optimized to identify the geometric parameters that minimize deflection under a 1 N palpation (normal direction) load at a bending angle of 90°. A Response Surface Methodology (RSM) was employed for this task. This class of Design of Experiments (DoE) approaches is particularly well-suited for systems exhibiting nonlinear behavior and computationally expensive evaluations (Zournatzis et al., 2023; Moutousi and Polygerinos, 2024), offering accurate response approximation with a reduced number of simulations. Specifically, a Box–Behnken design was selected. Three geometric features of the spine, groove depth, groove width, and vertebrae count, were identified as the most influential factors based on preliminary sensitivity analyses. Each factor was explored within a practically manufacturable range consistent with the actuator’s dimensional constraints (Table 4). The design necessitated 15 finite element simulations, comprising 13 unique parameter combinations and replicate center points to estimate experimental error.

**TABLE 4:** Design points for the response surface study.

Parameter	Low value	Mid value	High value
Groove width [mm]	0.4	0.6	0.8
Groove depth [mm]	0.4	0.7	1
Number of vertebrae	3	4	5

For each design point, the validated Finite Element Methods model was actuated to 90° and subjected to a 1 N palpation load; the resulting distal deformation was extracted as the response variable. A linear response surface was subsequently fitted to the dataset and evaluated for adequacy. The refined model retained groove width and groove depth, demonstrating moderate predictive capability (adjusted *R* ^2^ = 0.5378, Predicted $R^{2}$ = 0.3549, with satisfactory f and p-values). The number of vertebrae was deemed statistically insignificant within the studied domain. This lack of significance was unexpected, leading to the hypothesis of a potential performance interaction effect between vertebrae count and groove width. To investigate this, a secondary study utilizing a Central Composite Design (CCD) was conducted. With the groove depth fixed at its optimal value of 1 mm, nine unique combinations of groove width and vertebrae count were simulated. The results confirmed the statistical insignificance of the vertebrae count regarding the stiffness augmenting capabilities of the spine. The reason for this is that the vertebrae count in this range provides almost identical bending resistance between spines. Additionally, for the loads tested, the contact between vertebrae is practically rigid leaving only the factors that determine the contacts efficacy, groove width and depth, to affect the spine’s performance.

The optimization analysis revealed that groove depth exerted the dominant influence on structural performance, while groove width played a secondary yet statistically significant role. Conversely, vertebrae count was found to have negligible impact within the studied range. The optimal geometric configuration was defined by a groove depth of 1 mm and a groove width of 0.4 mm (Figures 5a,b), given the insensitivity to vertebrae count, any value between 3 and 5 is permissible. This configuration achieved an absolute stiffness improvement of 800% relative to the baseline Control actuator evaluated in preliminary experiments. The response surface predictive model produced absolute stiffness improvement between 240% and 800%, confirming that systematic parameter tuning can significantly enhance load-bearing capacity without compromising manufacturability or bending compliance, while facilitating modular stiffness tuning across a broad operational range.

## 4 Actuation unit and control

**FIGURE 6:**
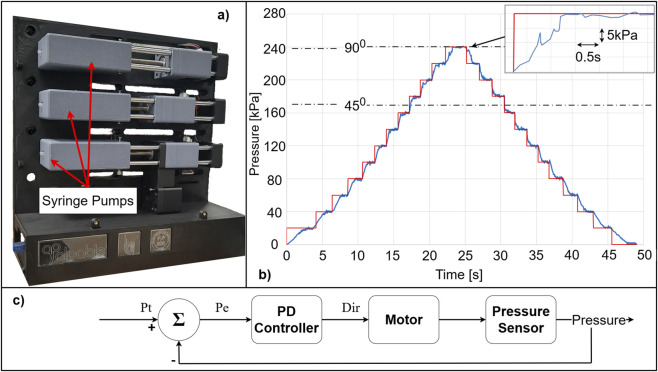
**(a)** Complete pump system that supports 3 pneumatic supply modules. **(b)** Measured pressure response of the pump (blue) in comparison the requested pressure (red), while inflating and deflating the actuator from 0° to 90° and back, at 20 kPa increments with 1s stasis at each pressure level; steady state error of 0.5 kPa at the most demanding pressure of 240 kPa. **(c)** Closed loop PD control schematic of the pump.

To drive the soft bending actuator safely, compactly, and repeatably for Minimally Invasive Surgery (MIS), a syringe pump (Figure 6a) was developed to avoid bulky compressors and complex valves while inherently limiting the maximum air volume released into the patient cavity in a failure scenario (60 mL at 1atm). Additionally, the system allows continuous, high-resolution pressure modulation, providing a smooth response free from the discrete switching and ripples common in PWM solenoid systems.

The pump uses a lead screw driven linear actuator. Unlike conventional designs with a stationary motor and separate carriage, the motor here translates with the lead screw. The compliant plunger shaft was replaced with a rigid 3D-printed interface that directly engages the syringe seal, reducing footprint and compliance. The design is scalable for 20–60 mL syringes, allowing selection of pressure–volume envelopes to meet specific constraints.

Actuation is controlled via a proportional–derivative (PD) loop on the measured pressure (Figure 6c). Hardware constraints limit the rise time to 8.5 s ensuring compatibility with quasi-static palpation tasks and prioritizing safety and precision over speed, effectively eliminating any hysteresis introduced by the non linear nature of the actuator. In addition to that, the pump’s direct drive operation principle, ensures that at such speeds air moves slowly leading to negligible pressure reading errors due to high flow. Pressure resolution is 0.5 kPa, which corresponds to a resolution of 0.1° for the actuator bending angle and 0.25 mm displacement in tip position, supporting repeatable positioning and robust closed loop control. Pressure tracking experiments using stepped references across the full actuation range confirmed stable convergence, minimal overshoot, and consistent steady state error (Figure 6b).

## 5 Shape proprioception

### 5.1 Hardware setup and data acquisition

The experimental framework utilizes a synchronized multi-modal sensing environment for high-fidelity proprioceptive estimation. Each FBG sensor was monitored via a single-channel interrogator with a spectral resolution of 2 p.m. (Componous, FBGI, Florina, Greece), measuring both the wavelength and light intensity transmitted by each sensor during bending. Pneumatic excitation was controlled by the piston pump assembly (Section 4), with pressure data serially transmitted by a microcontroller. Spatial ground truth was provided by the motion capture setup described in Section 3.3. To ensure precise sensor-fusion, all subsystems (pressure, FBG, and motion capture) were interfaced via Robot Operating System (ROS) nodes sharing a unified master clock for synchronized time-stamping. Finally, the actuator underwent three inflation-deflation cycles up to 90° (240kPa) , with high frequency sampling to capture the continuous deformation manifold for both predictors and observations.

### 5.2 Learning architecture and sensor fusion

**FIGURE 7:**
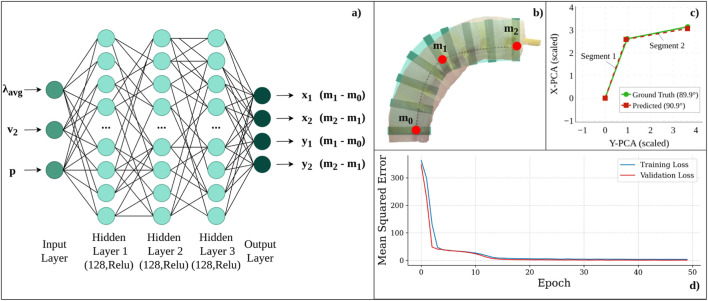
**(a)** The MLP model takes as input the average FBG wavelength $\left(\lambda_{\mathrm{avg}}\right)$ , transmitted light intensity $\left(v_{2}\right)$ , and pressure (p) , processes these through three hidden layers and outputs the relative spatial displacements ( x , y ) between the three markers $\left(m_{0},m_{1},m_{2}\right)$ . **(b)** A qualitative overlay of the MLP-predicted shape onto the physical soft robotic probe during a bending actuation at 90° . **(c)** A 2D quantitative comparison of the Ground Truth (green, solid) and the Predicted (red, dashed) marker positions at a maximum bending angle (∼90°) . **(d)** Learning curves illustrating the Mean Squared Error (MSE) over 50 epochs.

The input space fuses multi-modal optical and pneumatic data. As illustrated in the network architecture (Figure 7a), the proprioceptive model is implemented using a Multi-Layer Perceptron (MLP), Rumelhart et al. (1986), featuring three hidden layers of 128 neurons each with ReLU activations. The model utilizes a three-dimensional input vector $x={\left[\lambda_{\mathrm{avg}},\nu_{2},p\right]}^{T}$ , consisting of the average Fiber Bragg Grating (FBG) wavelength, the transmitted light intensity, and the real-time pneumatic pressure. All features are normalized against their operational limits to ensure training stability. To define the regression targets, the 3D tracking marker coordinates are projected onto their primary 2D bending plane using Principal Component Analysis (PCA), thereby mitigating out-of-plane noise. Furthermore, to isolate the actuator’s intrinsic shape deformation from absolute spatial referencing, the targets are formulated as relative distance vectors between the sequential markers: $\Delta {\vec{m}}_{1}={\vec{m}}_{1}-{\vec{m}}_{0}$ and $\Delta {\vec{m}}_{2}={\vec{m}}_{2}-{\vec{m}}_{1}$ , where ${\vec{m}}_{i}\in R^{2}$ represents the projected spatial coordinates of the $i_{th}$ marker. To facilitate localized error analysis along the reconstructed continuous shape, the actuator’s tracking data is evaluated across two discrete spatial segments defined by the physical marker placements, rather than being approximated as a rigid two-link kinematic model (Figure 7c). Segment 1 (Proximal) is defined by the relative vector between the base and the medial marker $\left({\vec{m}}_{1}-{\vec{m}}_{0}\right)$ , representing the deformation of the section closest to the pneumatic inlet. Segment 2 (Distal) is defined by the vector between the medial and tip markers $\left({\vec{m}}_{2}-{\vec{m}}_{1}\right)$ , capturing the curvature of the probe’s end section.

The dataset is partitioned by independent experimental trials rather than random shuffling to ensure robust evaluation. The network is trained for 50 epochs with a batch size of 32 and a learning rate of 0.001, with final model weights selected based on the minimum validation loss (Figure 7d). Model accuracy was evaluated using a relative Root Mean Square Error (RMSE) metric, normalized against the average $L^{2}$ norm of the ground truth spatial coordinates. To reconstruct the actuator’s geometry, the predicted waypoints were interpolated using Natural Cubic Splines to ensure C^2^ continuity along the skeletal centerline. This formulation preserves the continuously deformable nature of the manipulator while enabling reconstruction of both the end-effector position and orientation from the spline tangent at the distal tip. As illustrated in Figure 7b, the final body visualization was generated by aligning surface edges and cross-sectional rings via orthogonal offsets along the spline, producing a high-fidelity representation of the actuator’s deformed shape.

### 5.3 Performance evaluation and results

The efficacy of the multi-modal MLP in reconstructing the actuator’s shape was validated using the unseen test trials. The model’s performance was analyzed both through global coordinate regression and physically interpretable metrics, including Euclidean distance error, angular deviation of the two segments, and the total bending angle. The proprioceptive model achieved an overall parameter Root Mean Square Error (RMSE) of 0.52 across the continuous dataset. In kinematic modeling, it is standard practice to normalize the spatial RMSE by the system’s characteristic length, $L_{c}$ to provide a scale-invariant evaluation and contextualize errors physically. By defining the actuator’s instantaneous physical length as the characteristic length $L_{c,i}$ at each evaluated kinematic state i , the Normalized Root Mean Square Error (NRMSE) was calculated across all N sampled states as follows:

$$\text{NRMSE}=\frac{1}{N}\overset{N}{\underset{i=1}{\sum }}\left(\frac{{\text{RMSE}}_{i}}{L_{c,i}}\right)\times 100\%$$ (1)

By applying this state-by-state physical normalization, the model demonstrated an NRMSE of 1.01% (Equation 1).

To assess the practical utility of the sensing system for surgical or navigation tasks, the errors were decomposed into Euclidean distance (mm) and angular deviation (°) for each segment, alongside the global end-effector position and orientation. As shown in (Table 5), for the proximal section (Segment 1), the model achieved a Euclidean Mean Absolute Error (MAE) of 0.82 mm and an angular MAE of 1.43° . For the distal section (Segment 2), the MAE was 0.45 mm with a corresponding angular deviation of 0.56° . The global bending accuracy, represented by the Bending Angle metric, showed a mean error of 0.58° . A key requirement for soft surgical probes is reliability during extreme deformations. At a 90° bend configuration, end-effector positioning error (Tip metric) remained low at 0.79 mm, while the bending angular error was 0.95° .

**TABLE 5:** Shape reconstruction error metrics.

Metric	Prox. (S1)	Dist. (S2)	Tip	Bending angle
Euclidean MAE (mm)	0.82	0.45	1.13	–
Euclidean @ 90 ° (mm)	0.59	0.62	0.79	–
Angular MAE (°)	1.43	0.56	–	0.58
Angular @ 90 ° (°)	0.79	1.14	–	0.95

## 6 Conclusions and discussion

The bio-inspired stiffening spine significantly enhanced the PALPABLE soft bending actuator’s performance, achieving substantial stiffness gains without compromising compliance, manufacturability, or actuation efficiency.

Both Configuration 1 (grooved) and Configuration 2 (tendon-tensioned) outperformed the Control across all angles (0°, 45°, 90° ), loading directions (normal, lateral, soft lock), and load ranges. Notably, Configuration 1 increased stiffness by 800% at a 90°, satisfying the critical requirement of < 1 mm deformation under a 1 N load.

The Configuration 1 FEM model closely matched experimental data, showing maximum tip deviations of 0.9 mm (free-space) and 0.6 mm (loaded). Parametric optimization through Design of Experiments (DoE) identified groove depth as the dominant factor for stiffness, while vertebral count was statistically insignificant. This confirms that stiffness relies on contact efficacy rather than segment quantity. Ultimately, predictive models validated the architecture’s tunability, demonstrating stiffness increases between 240% and 800%.

A custom syringe pump provided repeatable PD pressure control with an error of 0.5 kPa. Its inherently limited air volume ensures patient safety, while high-pressure resolution enables precise positioning (0.25 mm and 0.1° errors). This modular architecture overcomes material-based stiffening limits, facilitating application-specific stiffness tuning and sensor integration.

Finally, combining FBG optical data (wavelength and intensity) with real-time pressure inputs via a data-driven MLP architecture achieved a high-fidelity shape proprioception. This multimodal model yielded a 1.01% NRMSE, with distal errors of just 0.79 mm and 0.95° at 0.90° bend. These results demonstrate the sub-millimeter accuracy required for sensitive surgical manipulation. Future work will explore dynamic loading, fatigue behavior, and closed-loop force control using real-time feedback.

## Data Availability

The datasets presented in this article are not readily available because of patent submission considerations. Requests to access the datasets should be directed to Panagiotis Polygerinos, polygerinos@hmu.gr.
